# Resveratrol attenuates cortical neuron activity: roles of large conductance calcium-activated potassium channels and voltage-gated sodium channels

**DOI:** 10.1186/s12929-016-0259-y

**Published:** 2016-05-21

**Authors:** Ya-Jean Wang, Ming-Huan Chan, Linyi Chen, Sheng-Nan Wu, Hwei-Hisen Chen

**Affiliations:** Center for Neuropsychiatric Research, National Health Research Institutes, 35, Keyan Road, Zhunan, Miaoli County 35053 Taiwan; Institute of Neuroscience, National Chengchi University, 64, Sec.2, ZhiNan Road, Wenshan District, Taipei City, 11605 Taiwan; Research Center for Mind, Brain, and Learning, National Chengchi University, 64, Sec.2, ZhiNan Road, Wenshan District, Taipei City, 11605 Taiwan; Institute of Molecular Medicine, National Tsing Hua University, 101, Section 2, Kuang-Fu Road, Hsinchu, 30013 Taiwan; Department of Physiology, National Cheng Kung University Medical College, 1 University Road, Tainan City, 70101 Taiwan; Department of Pharmacology and Toxicology, School of Medicine, Tzu Chi University, 701, Section 3, Chung-Yang Road, Hualien, 97004 Taiwan

**Keywords:** Resveratrol, BK_Ca_ channel, Sodium channel, Action potential, Firing rate

## Abstract

**Background:**

Resveratrol, a phytoalexin found in grapes and red wine, exhibits diverse pharmacological activities. However, relatively little is known about whether resveratrol modulates the ion channels in cortical neurons. The large-conductance calcium-activated potassium channels (BK_Ca_) and voltage-gated sodium channels were expressed in cortical neurons and play important roles in regulation of neuronal excitability. The present study aimed to determine the effects of resveratrol on BK_Ca_ currents and voltage-gated sodium currents in cortical neurons.

**Results:**

Resveratrol concentration-dependently increased the current amplitude and the opening activity of BK_Ca_ channels, but suppressed the amplitude of voltage-gated sodium currents. Similar to the BK_Ca_ channel opener NS1619, resveratrol decreased the firing rate of action potentials. In addition, the enhancing effects of BK_Ca_ channel blockers tetraethylammonium (TEA) and paxilline on action potential firing were sensitive to resveratrol. Our results indicated that the attenuation of action potential firing rate by resveratrol might be mediated through opening the BK_Ca_ channels and closing the voltage-gated sodium channels.

**Conclusions:**

As BK_Ca_ channels and sodium channels are critical molecular determinants for seizure generation, our findings suggest that regulation of these two channels in cortical neurons probably makes a considerable contribution to the antiseizure activity of resveratrol.

## Background

Resveratrol (trans-3,4',5-trihydroxystilbene), a polyphenolic phytoalexin, is derived from some edible materials, including grape skins, peanuts, red wine, and other berries. It has been demonstrated that resveratrol displays diverse pharmacological activities, including anti-platelet [2], anti-carcinogenic [4], anti-viral [9, 18, 27] and cardio-protective effects [32, 33, 37, 43, 47]. Moreover, there is accumulating evidence indicating that resveratrol exhibits neuroprotective effects [8, 11, 23, 28]. For example, resveratrol attenuates kainic acid-mediated convulsions and the associated neurotoxicity [16, 42, 49, 54] and also protects against pentylenetetrazole-induced seizures [31, 40]. Furthermore, resveratrol has the ability to inhibit the electrical activity of neurons [26, 30, 50], enabling this compound ideal as a neuroprotective agent against excitatory effects on neurons. This compound can inhibit neuronal discharges in rat hippocampal CA1 area [25] and suppress epileptiform discharges mediated by glutamate [25]. In addition, resveratrol has the ability to produce a dose-dependent inhibition of field excitatory postsynaptic potentials [11]. These effects are likely associated with the alterations in neuronal cell membrane ion channel activities.

In fact, resveratrol has been reported to regulate ion channel activities in a variety of cells. For example, resveratrol inhibits K_ATP_ currents [7], L- and T-type Ca^2+^ currents and swelling-dependent Cl^−^ currents evoked by either hypotonicity or high extracellular glucose-ion conductances in insulin secreting cells [20]. Voltage-gated sodium channels in cardiomyocytes [47] and rat dorsal root ganglion neurons [21] are also blocked by resveratrol. In contrast, resveratrol stimulates BK_Ca_ channels in vascular endothelial cells [24] and human cardiac fibroblasts [51].

Despite this, the effects of resveratrol on the membrane properties and the ion channels of neurons have not yet been fully determined. Therefore, the present study examined the effects of resveratrol on BK_Ca_ currents and voltage-gated sodium currents by a voltage clamp setup in cortical neurons. Moreover, the effects of resveratrol on action potential firing rate and the BK_Ca_ channel inhibitor TEA (or paxilline)-induced hyperexcitability were also evaluated. Our data demonstrated that resveratrol activated BK_Ca_ channels, but inhibited voltage-gated sodium currents. Moreover, the action potential firing rates evoked by the depolarizing current and BK_Ca_ channel blockers were remarkably decreased by application of resveratrol. These results suggest that alterations of BK_Ca_ channel activity and sodium currents by resveratrol may contribute to its reducing effect on action potential firing rates of cortical neurons.

## Methods

### Cells preparations

All experiments were performed in accordance with the Laboratory Animal Center of National Tsing Hua University (NTHU) guidelines for the care and use of animals. Animal use protocols were approved by the NTHU Institutional Animal Care and Use Committee (Approval number 10126). The cerebral cortex was dissected from embryonic day 18 (E18) embryos of Sprague-Dawley rats (purchased from BioLASCO Co., Ltd.). Then, the cells were treated with papain (10 U/mL). Dissociated cells were washed with PBS three times and re-suspended in minimal essential medium (MEM) supplemented with 5 % HS and 5 % FBS. Cells were seeded onto 30 μg/ml poly-L-lysine-coated coverslips and then cultured in neurobasal medium with B27 (containing additional 25 μM glutamate) on DIV (day in vitro) 1. On DIV 3, cells were treated with 5 μM cytosine 1-β-D-arabinofuranoside. Half of the neurobasal and glutamine media were replaced by fresh media every 3 days. Glial contamination of neuronal cultures was consistently less than 5 % on DIV7.

The clonal strain, HCN-1A cell line (CRL-10442), originally derived from a cortical tissue removed from a patient undergoing hemispherectomy for intractable seizures, was obtained from the American Type Culture Collection (ATCC). The cells were cultured at 37 °C in a humidified atmosphere of 5 % CO_2_ and 95 % air. Culture media (e.g., Dulbecco's Modified Eagle Medium (Life Technologies), were supplemented with 20 % heat-inactivated fetal bovine serum, 1 % P/S, and 2 mM L-glutamine (Life Technologies) [52].

### Chemicals and solutions

Resveratrol (purity ≥99 %), NS1619 (purity ≥ 99 %), tetrodotoxin (TTX) (purity ≥98 %), tetraethylammonium chloride (TEA) (purity ≥98 %), paxilline, a mycotoxin of penicillium origin (purity ≥98 %), and papain (purity ≥99 %) were purchased from Sigma-Aldrich. Resveratrol and NS1619 were dissolved in dimethylsulfoxide (DMSO). TTX was dissolved in water. All culture media, FBS, HS, L-glutamate, trypsin/EDTA, and penicillin-streptomycin were purchased from Invitrogen. The composition of normal Tyrode's solution was as follows (in mM): NaCl 136.5, KCl 5.4, CaCl_2_ 1.8, MgCl_2_ 0.53, glucose 5.5, and HEPES 5.5 (pH 7.4). To record BK_Ca_ currents and action potentials, the patch pipettes were filled with a solution (in mM): KCl 145, MgCl_2_ 1, Na_2_ATP 3, EGTA 0.1, and HEPES 5.5 (pH 7.2). To measure *I*_Na_, potassium ions inside the pipette solution were replaced with equimolar Cs^+^ ions (pH 7.2). In single-channel current recordings of BK_Ca_ channels, the high K^+^-bathing solution contained (in mM): KCl 145, MgCl_2_ 0.53, CaCl_2_ 1.8, and HEPES 5 (pH 7.4). The pipette solution contained (mM): KCl 145, MgCl_2_ 2, and HEPES 5 (pH 7.2).

### Electrophysiological recordings and data analysis

Membrane currents and action potentials of primary embryonic rat cortical neurons were recorded in the whole-cell configuration of the patch-clamp technique using patch pipettes with a tip resistance of 3–6 MΩ, unless mentioned otherwise. All analog signals were filtered at 1 or 3 kHz before digitization at 10 or 50 kHz and stored on a hard disk using a PC-compatible computer. All data analysis was performed with Clampfit software (Molecular Devices). The EPC-10 amplifier was used for voltage-clamp recording and current-clamp recording [36, 56]. The signals recorded from human cortical (HCN-1A) neurons were stored in a Slimnote VX_3_ computer (Lemel) via a universal serial bus port at 10 kHz through a Digidata 1322A interface (Molecular Devices). This device was controlled by the pCLAMP 9.0 software (Molecular Devices). The signals were low-pass filtered at 1 to 3 kHz. Ion currents recorded during cell-attached recordings were stored and analyzed using the pCLAMP 9.0 software (Molecular Devices), the Origin 7.5 software (Microcal Software, Inc), the SigmaPlot 7.0 software (SPSS, Inc), or custom-made macros in Excel 2003 (Microsoft).

The PatchMaster-generated voltage-step protocols were employed to investigate the current-voltage (*I-V*) relations for ion currents in embryonic rat cortical neurons. To calculate percentage inhibition of resveratrol on *I*_Na_, the cells were depolarized from a holding potential of −80 mV, and a 50-msec depolarizing pulse to −20 mV. The amplitude of *I*_Na_ obtained at the level of −20 mV was then compared after addition of the different concentrations (5–40 μM) of resveratrol. The amplitude of *I*_Na_ in the presence of 0.1 % DMSO was taken as 100 %. Then, those exposed to different concentrations of resveratrol were then compared. In these experiments, the TTX was taken as a positive control. The BK_Ca_ channel currents in response to resveratrol were examined in the condition of the extracellular solution containing TEA (1 mM), a BK_Ca_ channel blocker, to block BK_Ca_ channel currents. The net currents subtracted before and after treatment with BK_Ca_ channel blockers were defined as BK_Ca_ channel currents. For these experiments, the cells were depolarized from −50 to +70 mV. The amplitude of potassium outward current was measured at the end of the depolarizing pulses. The NS1619, a BK_Ca_ channel activator, was taken as a positive control. In single-channel recordings, open probability (N · P_o_) and single-channel conductance for BK_Ca_ channels were determined by all-point amplitude histograms. Open lifetime distributions were fitted with logarithmically scaled bin width. In cell-attached configuration, the relationships between membrane potentials and the probability of channel openings were fitted with a Boltzmann function of the form: N · P_o_ = n_P_/{1 + exp[−(V − V_1/2_)/k]} where n_P_ is the maximal open probability, V is the membrane potential in mV, V_1/2_ is the voltage at which there is half-maximal activation, k is the slope factor of the activation curve.

### Statistical analysis

Results were expressed as mean ± standard error (*n* = number of patches or cells). The significance of differences between means was tested with paired *t*-test and differences were considered significant at *P* < 0.05.

## Results and Discussion

**Fig. 1:**
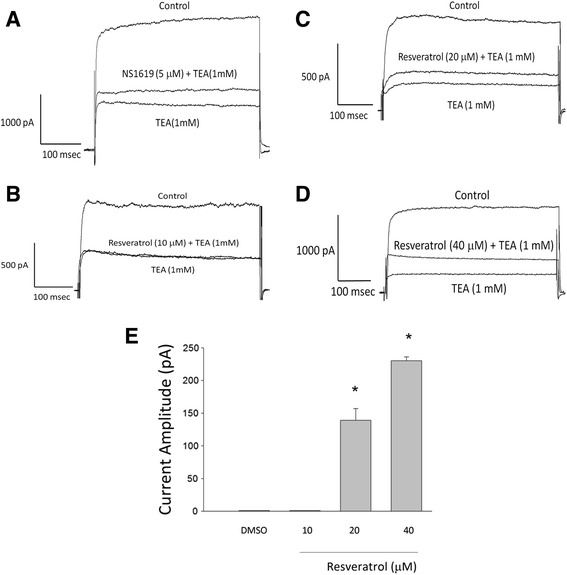
Stimulatory effect of resveratrol on TEA-sensitive potassium currents in embryonic rat cortical neurons. The whole-cell recording was conducted in these experiments. Because BK_Ca_ channels are typically blocked by externally applied tetraethylammonium (TEA, 1 mM), this compound is often used to determine the contribution of BK_Ca_ channels to the whole-cell currents. All cells were held at -50 mV in Tyrode’s solution containing 1.8 mM CaCl_2_. Then, cells were depolarized from −50 to +70 mV with a duration of 300 msec. **a** The currents were recorded in the presence of TEA (1 mM) combined with NS1619 (5 μM) from the same cell. **b**, **c**, **d** Original current traces are representative of 3 experiments. The currents were recorded in the presence of TEA (1 mM) or TEA (1 mM) combined with resveratrol (10, 20 and 40 μM) from the same cell. Resveratrol (20 and 40 μM, but not 10 μM) increased the TEA-sensitive current in these cells (*n* = 3). **e** Bar graph showing the effect of resveratrol on TEA-induced currents. Each bar indicates the mean ± SEM. (*n* = 3) ^*^Significantly different from control (*P* < 0.01)

The large conductance Ca^2+^-activated K^+^ (BK_Ca_) channel currents in response to resveratrol were examined in the condition of the intracellular dialysis with solution containing EGTA 0.15 mM and the extracellular solution containing CaCl_2_ 1.8 mM. The potassium outward currents were elicited by 300 ms depolarization of membrane potential to +70 mV from holding potential −50 mV. To evaluate whether resveratrol could affect the BK_Ca_ currents in cortical neurons, the effects of resveratrol on the current amplitude were examined in the presence of TEA, a BK_Ca_ channel blocker. The net response subtracted before and after treatment with TEA, was defined as BK_Ca_ channel current. A putative BK_Ca_ channel activator NS1619 was used as a positive control. The current difference between application of TEA, combination of NS1619 and TEA (Fig. 1a), or combination of resveratrol and TEA (Fig. 1b–d) in the same cell was the component of activated BK_Ca_ currents. TEA-sensitive K_Ca_ currents recorded at +70 mV were not significantly altered after exposure to resveratrol at 10 μM (Fig. 1b), whereas higher concentrations of resveratrol (20 and 40 μM) enhanced TEA-sensitive K_Ca_ currents. The peak outward currents recorded at +70 mV before and after exposure to resveratrol (20 μM) were 139 ± 18 pA and 230 ± 6 pA (*n* = 3), respectively (Fig. 1c). Resveratrol (40 μM) further enhanced TEA-sensitive K_Ca_ currents. The peak outward currents recorded at +70 mV were 254 ± 12 pA and 622 ± 13 pA (*n* = 3), respectively (Fig. 1d).

The BK_Ca_ channels are both voltage-gated and intracellular Ca^2+^ dependent [41], which are sensitive to TEA or paxilline [38]. When activated by cell membrane depolarization and elevation of intracellular Ca^2+^ concentration, BK_Ca_ channels allow the efflux of K^+^ out of the cell, thus repolarizing and hyperpolarizing the membrane potential. This turns off voltage-dependent Ca^2+^ channels and thus inhibits the influx of Ca^2+^ into the cell. These negative feedback mechanisms allow BK_Ca_ channels to play an important role in regulating firing properties. BK_Ca_ channels are expressed in various brain neurons where they play important roles in regulating action potential duration, firing frequency and neurotransmitter release [34]. The present study revealed that resveratrol enhanced TEA-sensitive potassium currents, suggesting that it is capable of stimulating BK_Ca_ currents in cortical neurons.

**Fig. 2:**
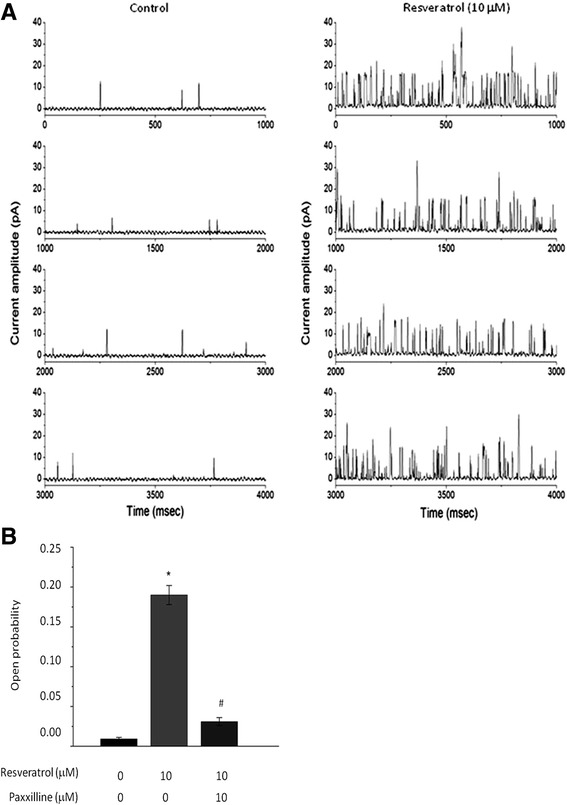
Resveratrol evoked the BK_Ca_ channel responses in human cortical (HCN-1A) neurons. In these experiments, cells were bathed in high-K^+^ solution and cell-attached current recordings were made. The holding potential was constantly set at +60 mV. **a** Original current trace obtained in the absence (left) and presence (right) of 10 μM resveratrol. Resveratrol was applied to the bath solution. The upward deflection indicates the opening events of the channels. **b** Bar graph showing the effect of resveratrol and resveratrol plus paxilline on the open probability of BK_Ca_ channels. Each bar indicates the mean ± SEM (*n* = 7-9). Res: 10 μM resveratrol; Pax: 1 μM paxilline. ^*^Significantly different from control (*P* < 0.05). ^#^Significantly different from resveratrol alone group (*P* < 0.01)

To further elucidate the effect of resveratrol on the activity of BK_Ca_ channels, the single-channel recording with a cell-attached configuration was performed in human cortical neurons. These studies were performed in symmetrical K^+^ (145 mM) concentration and the bath solution contained 1.8 mM Ca^2+^. The cells were held at +60 mV. As shown in Fig. 2a, when resveratrol (10 μM) was applied to the chamber, the activity of channel openings was significantly increased. However, no change in single-channel amplitude was demonstrated in the presence of resveratrol. These findings suggested that its binding site should not be located in the pore region of the BK_Ca_ channels. In addition, resveratrol-induced changes in the probability of channel openings were reversed by a BK_Ca_ channel blocker paxilline (1 μM) (Fig. 2b), revealing that the components affected by resveratrol are mediated by its action on BK_Ca_ channels.

**Fig. 3:**
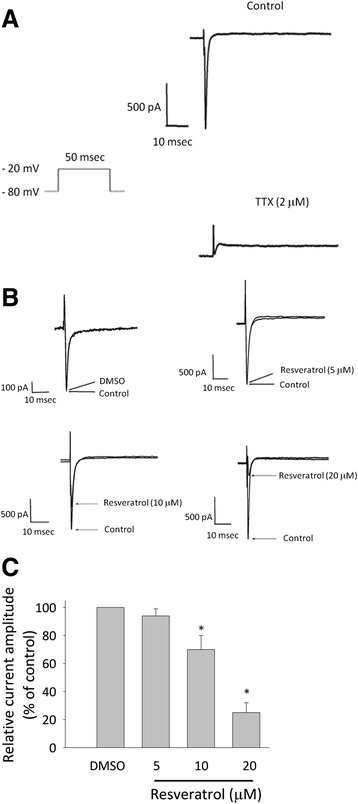
Concentration-dependent effect of resveratrol on voltage-gated sodium currents. The cells were bathed in Ca^2+^-free, Tyrode's solution. **a** Functional expression of the voltage-gated sodium current in embryonic rat cortical neurons. Superimposed current traces in control (top panel) and during exposure to 2 μM TTX (lower panel). Under the experimental condition, depolarizing voltage command from a holding potential of −80 mV elicited an inward current sensitive to TTX. This inward Na^+^ current was maximally activated by a test pulse at −20 mV (*n* = 8). **b** Concentration dependent effects of resveratrol on inward Na^+^ currents. The currents were evoked depolarizing the cells from a holding potential of −80 mV to −20 mV. Representative traces showing the depression of inward Na^+^ currents by resveratrol at different concentrations in these cells. **c** The graph shows the concentration dependent effect of resveratrol on the amplitudes of the inward currents measured at the peaks (*n* = 3-5). ^*^Significantly different from control group (*P* < 0.05)

Sodium currents were evoked by a depolarizing pulse to −20 mV from a holding potential of −80 mV. Being a positive control, TTX, a voltage-gated sodium channel blocker, significantly reduced sodium current amplitude (Fig. 3a). Resveratrol (10 and 20 μM) significantly decreased sodium current amplitude at the level of −20 mV within 5 min, whereas resveratrol (5 μM) did not affect the amplitude of sodium currents (Fig. 3b). To calculate the percentage inhibition of resveratrol on sodium currents, the cells were depolarized from −80 to −20 mV with a duration of 50 ms and the peak amplitude of sodium inward currents was measured. The amplitude of sodium currents in the control condition was taken as 100 % and those exposed to different concentrations of resveratrol were then compared. Fig. 3c illustrated that resveratrol (5–20 μM) reduced the amplitude of sodium currents in a concentration-dependent manner.

**Fig. 4:**
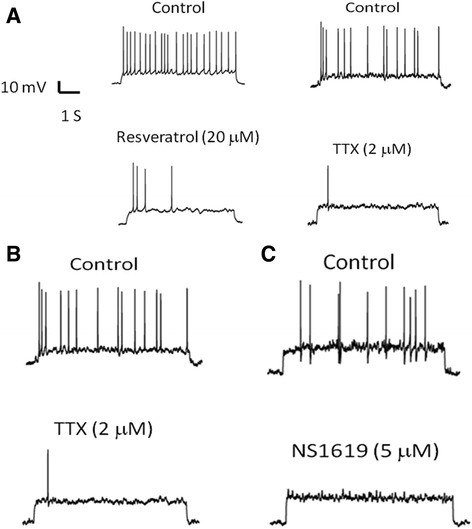
Resveratrol reduced action potential firing in embryonic rat cortical neuron. Current-clamp configuration was made in these experiments. All cells were bathed in Tyrode’s solution containing 1.8 mM CaCl_2_. Trains of action potentials were evoked by a depolarizing current step. **a** Original potential traces obtained in control (upper) and in the presence (lower) of resveratrol (20 μM) by injecting a threshold current. **b** The potential traces obtained in the absence and presence of TTX (2 μM). **c** The potential traces obtained in the absence and presence of NS1619 (5 μM). In the presence of resveratrol, TTX, and NS1619, the action potential firing frequency was significantly decreased

In fact, the TTX-S sodium currents and BK_Ca_ currents are important in shaping the action potential of neurons [3, 13]. The effects of resveratrol on cellular excitability were examined in rat cortical neurons with repetitive firings evoked by positive current injection. For the measurement of evoked action potential firings in current-clamp, the membrane potentials were held at −60 mV. During a 6 s injection of a positive current (ranging from 5 to 30 pA), repetitive firings could be evoked in these cells. The frequency (Hz) of action potential firings was determined by dividing the number of action potentials by the duration of the recording period. Resveratrol (20 μM) was applied into the chamber for 2 min and reduced the action potential firing frequency from the control value of 6.9 ± 0.3 Hz to 0.4 ± 0.2 Hz (*n* = 8) (Fig. 4a). Similarly, when the cells were treated with TTX, a specific sodium channel blocker, or BK_Ca_ channel activator NS1619 (5 μM), the frequency of action potentials was significantly decreased in embryonic rat cortical neurons (Fig. 4b, c).

**Fig. 5:**
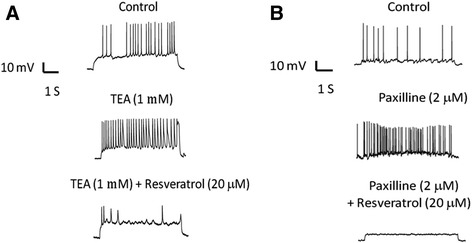
Resveratrol reduced TEA- or paxilline-evoked increases in action potential firing in embryonic rat cortical neuron. **a** Action potential firing was measured in control, in the presence of TEA (1 mM), and TEA combined with resveratrol (20 μM). **b** Action potential firing was measured in control, in the presence of paxilline (20 μM), and paxilline combined with resveratrol (20 μM). TEA and paxilline significantly increased the action potential firing frequency. Upon application of 20 μM resveratrol in the presence of TEA and paxilline, the excitability was suppressed significantly

The effects of resveratrol on action potential firings were also examined in the presence of two BK_Ca_ channel blockers, TEA or paxilline, to evaluate whether resveratrol could suppress the increased excitability of cortical neurons evoked by inhibition of BK_Ca_ channels. As shown in Fig. 5a, application of TEA increased the action potential firing rate from the control value of 3.5 ± 0.3 to 5.7 ± 0.7 Hz. In the presence of 20 μM resveratrol combined with TEA, the increase in firing rate evoked by TEA was reduced to 0.1 ± 0.1 Hz. In addition, when the cells were treated with paxilline, the action potential firing rate was also increased. After applications of resveratrol combined with paxilline, the increase in firing rate evoked by paxilline was significantly reduced (Fig. 5b). These results suggest that BK_Ca_ channel opening and sodium channel inhibition by resveratrol could underlie, at least in part, the inhibition of action potential firing in the cortical neurons.

Resveratrol has been reported to stimulate BK_Ca_ currents in human vascular endothelial cells and human cardiac fibroblasts [24, 51], which might be associated with its cardioprotective effect. The present study demonstrated that resveratrol could stimulate the activities of BK_Ca_ channels in cortical neurons. In fact, BK_Ca_ channel is considered to be one of the intrinsic molecular determinants for the control of neuronal excitability in the central nervous system and play a role in the etiology of some neurological diseases. Recent studies have demonstrated the implication of BK_Ca_ channels in Fragile X Syndrome (FXS) pathology [22]. In fact, a selective BK_Ca_ channel opener molecule (BMS-204352) rescues a broad spectrum of behavioral impairments (social, emotional and cognitive) in an animal model of FXS [17]. Resveratrol might be also beneficial to patients with FXS.

BK_Ca_ channels also play an important role in seizure etiology. Loss-of-function BK_Ca_ channel mutations can lead to temporal lobe epilepsy, tonic-clonic seizures and alcohol withdrawal seizures [34, 35]. Paradoxically, some mutations in BK_Ca_ channel subunit can give rise to channel gain-of-function that leads to development of idiopathic epilepsy (primarily absence epilepsy) [34]. Thus, both loss-of-function and gain-of-function BK_Ca_ channels might serve as molecular targets for drugs to suppress certain seizure phenotypes including temporal lobe seizures and absence seizures, respectively. Actually, resveratrol has been found to reduce the kainate-induced temporal lobe epilepsy [16, 54], suggesting that resveratrol might have potential for treatment of this seizure type through activation of BK_Ca_ channels.

There are nine recognized members of the voltage-gated sodium channel family (Na_v_1.1–Na_v_1.9). Of these, Na_v_1.1, Na_v_1.2, Na_v_1.3 and Na_v_1.6 are highly expressed in the central nervous system [10]. In particular, the Na_v_1.1, Na_v_1.2 and Na_v_1.6 sodium channels are expressed in cortical tissue [55], which are all TTX-sensitive [5]. Consistently, we found that the sodium currents recorded in the rat cortical neurons are totally blocked by TTX. It appears that Na_v_1.1, Na_v_1.2 and Na_v_1.6 sodium channels should be the targets for resveratrol.

It has been shown that resveratrol suppresses the TTX-S sodium currents in rat dorsal root ganglion neurons [21] that plays an important role in pain transmission [1, 53]. Inhibition of sodium currents by resveratrol may account for its analgesic effects [12, 14, 44]. The present study demonstrated that resveratrol can inhibit the TTX-S sodium currents in rat cortical neurons. Several lines of evidence revealed that the pathophysiology of both acquired and inherited epilepsy is associated with abnormal expression or function of voltage-gate sodium currents [29]. The Na_v_1.1 or Na_v_1.2 mutations are associated with generalized epilepsy and inherited epilepsy [6, 19]. Together with the observations that the protective effects of resveratrol against seizure activities caused by kainic acid or pentylenetetrazole [16, 45, 46, 48], our findings suggest that in addition to activation of BK_Ca_ channels, blockade of voltage-gated sodium channels in the cortical neurons might also contribute to the anti-seizure effects of resveratrol.

## Conclusions

In summary, our results suggested that the suppressing effect of resveratrol on action potential firing rate may be mediated by opening BK_Ca_ channels and closing voltage-gated sodium channels. Current clinical available anti-epileptics are mostly sodium channel blockers. The sodium channel blockers were very effective for treating generalized epilepsy with febrile seizures plus, while it aggravates symptoms in patients with severe myoclonic epilepsy of infancy [15, 39]. With dual effects on BK_Ca_ and sodium channels, resveratrol might have the potential as a broad-spectrum anti-seizure medication.

## Abbreviations

BK_Ca_: large-conductance calcium-activated potassium channel
HCN-1A: human cortical neuron
*I*_Na_: sodium current
TEA: tetraethylammonium
TTX: tetrodotoxin
